# A systematic review of ENT retractions

**DOI:** 10.1007/s00405-024-08980-8

**Published:** 2024-10-14

**Authors:** Rosalind Di Traglia, Henry Dunne, James Tysome, Matthew E. Smith

**Affiliations:** 1 School of Clinical Medicine, University of Cambridge, Cambridge, UK; 2 Cambridge University Hospitals NHS Foundation Trust, Cambridge, UK; 3 Department of ENT, Addenbrookes Hospital, Cambridge University Hospitals NHS Foundation Trust, Cambridge, CB2 0SP UK

**Keywords:** Retracted, Retraction, Publications, Otolaryngology, ENT

## Abstract

**Purpose:**

Retraction is the removal of published material due to flaws in research that cannot be corrected. Our aim was to perform a systematic review of all retracted literature in Ear Nose and Throat to understand the characteristics of retraction and the citations of retracted literature.

**Methods:**

The Retraction Watch, EMBASE and MEDLINE databases were systematically searched to yield relevant retractions. Two independent authors performed abstract and full-text screening. Non-relevant texts, articles in non-English languages, and articles that were neither published (protocols) or retracted (expression of concern) were excluded.

**Results:**

We found 225 retractions in Ear Nose and Throat literature from 1992 to 2023. The number of retractions increased with time, and the average time-to-retraction was 1 year (range 0–29). Most articles were retracted due to misconduct (72%). In total, 191/225 of retractions were signposted with a retraction notice; 90.6% of notices were linked to the original manuscript; 96.9% specified the reason for retraction and 100% were freely accessible. Publications were cited more after retraction (median 2, range 0–215 vs median 0, range 0–78, *Z *–1.78, *p* = 0.075), however this was not significant, and appeared to improve with a shorter retraction time (RS 0.67, *p* < 0.001).

**Conclusion:**

Retractions, although rare, are increasing across all scientific disciplines. Our data suggests that retractions are both efficiently and transparently publicised in the Ear Nose and Throat Literature. Investigators should be veracious when conducting their own research and regularly appraise manuscripts to ensure that misinformation is not perpetuated, remaining aware that retracted articles continue to be cited.

**Supplementary Information:**

The online version contains supplementary material available at 10.1007/s00405-024-08980-8.

## Introduction

Study retraction is becoming increasingly apparent in the scientific body of literature. Retraction can be defined as the process of removing published material due to flaws in research that invalidate the author’s conclusions, for example honest errors and misconduct. A retraction can be requested by readers, editors, an institution, or by authors identifying errors in their own work. Publishers may initially issue an ‘expression of concern’ for articles undergoing an investigation into misconduct and/or those with inconclusive evidence from such investigations; the former may eventually be retracted. Publications containing minor offences are amenable to ‘correction’ instead of retraction [1]. Retractions are distinct from withdrawal as they occur prior to publication when authors remove their submission for any number of reasons, including concerns over peer review or journal integrity.

The rate of retracted articles indexed in PubMed increased from 0.002% in 1980 to 0.02% by 2009 [2]. The cause for the increasing incidence of retraction remains uncertain. It has been postulated that submitted manuscripts are undergoing more rigorous scrutiny or that there is a greater abundance of flawed research being published [3].

The Committee of Publication Ethics (COPE) provides a framework to guide editors issuing retractions for genuine mistakes and misconduct. It defines academic misconduct as any deviance in research practice that compromises the reliability of results. This may include plagiarism, falsified data, author dispute, failure to obtain ethics approval, manipulated peer review, failure to disclose conflict of interest, and duplicate publishing which refers to the practice of identical or overlapping article submissions by the same author [4].

Although retraction is a strategy to maintain scientific integrity, the reality is that retracting articles does not eliminate the risk of citing retracted literature. COPE guidelines have stated that journals must publicise a retraction notice that clearly defines the cause of retraction, and is prompt, freely accessible and linked to the original publication [1]. However, some retraction notices are hidden behind a paywall. Moreover, retraction notices may not be available in print publications, or they may be delayed after the paper has already been published.

Our aim is to evaluate retractions in Ear Nose and Throat (ENT) research, particularly the characteristics and causes of retractions, to increase our understanding of this issue. We will also explore the type of retraction notices, the presence of paywall, and citations of retracted articles, as unaware authors may continue to cite misinformation. It is imperative that research output is regularly scrutinised, particularly in surgery, where the dissemination of erroneous material prior to and after retraction could lead to significant patient harm [5].

## Methods

We performed a systematic review of retracted ENT literature in MEDLINE, EMBASE, and the Retraction Watch Database (retractiondatabase.org) on the 4th of September 2023. We included all basic science and clinical research from years 1946 till present in MEDLINE and from years 1974 till present in EMBASE. We searched articles from years 1986 till present in the Retraction Watch Database. All study types were included in the review. We performed the systematic review in concert with PRISMA guidance [6].

### Search methods and article screening

We developed several search strategies based on previous reviews of retracted surgical literature, as there is currently no validated search strategy. In MEDLINE, we performed word searches including the following keywords: “ear nose or throat”, “otolaryngology”, otorhinolaryngology”, “Ear Diseases”, “Pharynx”, “Nose Diseases “ENT” “head or neck” and then applied the ‘retracted publication’ or ‘retraction of publication’ limit to obtain retracted articles. Our search strategy for EMBASE consisted of similar combination of keywords and used the “Retraction Notice” filter instead (see supplemental material for all search strategies). We also obtained retracted papers from the Retraction Watch, a free database of retracted research, under the subject heading ‘Medicine- otorhinolaryngology’. Two authors independently conducted title and abstract screening on Rayyan software (https://www.rayyan.ai/). Unrelated subjects, duplicates, non-retracted papers and articles in non-English languages were excluded at this stage. A senior third reviewer was brought in to mediate any discrepancies and achieve consensus over selected articles.

### Data extraction

Two authors independently extracted data on article characteristics, such as publishing journal; journal impact factor; date of publication; date of retraction; country origin of publication and study design (e.g. basic science/translational or RCT). We subclassified content into subspecialties such as head and neck (H&N), rhinology, otology and paediatric before extracting data. We examined the type of retraction notice, and we evaluated their accessibility to the public with the absence of a paywall.

We identified the causes of retraction from the Retraction Watch database. For citations we obtained from EMBASE and MEDLINE, we used the publisher’s retraction notices and referenced COPE guidelines and the Retraction Watch to classify the reasons. We then grouped the causes of retraction as misconduct and non-misconduct related, so that results could be discussed in the context of existing literature that used the same categories. Article duplication, plagiarism, falsified data, manipulated peer review/ethical review and author dispute were grouped as misconduct related.

Furthermore, we conducted a citation analysis by evaluating the number of citations the article received before and after retraction in Web of Science. We also obtained Journal impact factor from Journal Citation Reports in Web of Science.

### Statistical analysis

Data analysis was performed in IBM SPSS Statistics (Version 27). In our descriptive statistical analysis, we calculated medians and ranges as data was not normally distributed. With the exclusion of a time series analysis, regression was not possible, as the relationship between variables was nonlinear and there was significant heteroskedasticity; this would have led to errors in analysis. Alternatively, we performed Spearman’s rank correlation between continuous variables as the data was not normally distributed.

Mann–Whitney *U* was used to explore potential associations between citation number and the presence of retraction notice, paywall and the cause of retraction. All categorical data was dichotomised as paywall vs no paywall, misconduct vs non-misconduct, retraction notice vs no retraction notice. All types of retraction notices, including a link to updated Cochrane review were grouped into having a retraction notice present. We also explored associations between time-to-retraction and cause of retraction with Mann–Whitney *U*. A Wilcoxon test was adopted to conduct citation analysis of manuscripts before and after retraction as the sample was paired and not normally distributed. A *p* value less than 0.05 was considered statistically significant.

## Results

**Fig. 1:**
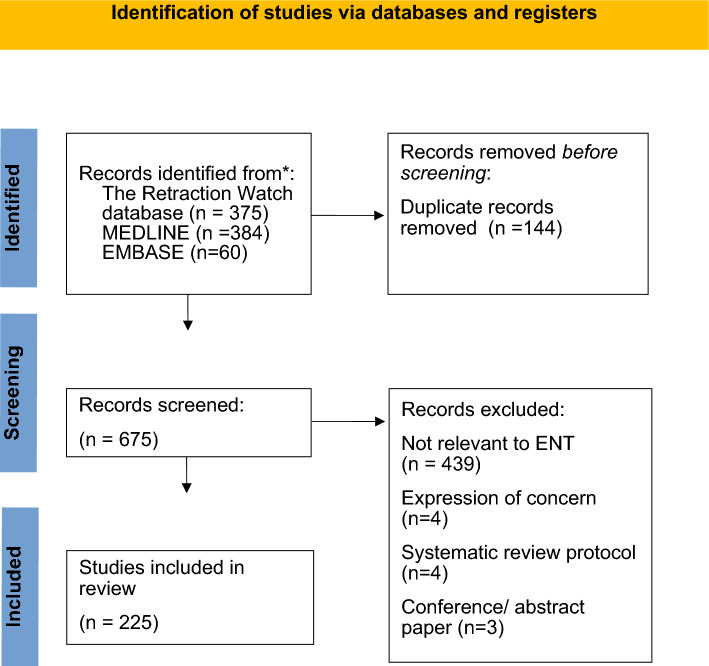
PRISMA flow chart of article screening and selection

Our systematic searches produced a total of 819 articles. We deduplicated the results and excluded 450 articles during title and abstract screening (see Fig. 1). A minority of studies were protocols (4), conference/abstract papers (3), or were publications that received expressions of concern (4). After screening, 225 papers were eligible for the review. Most of the studies were excluded because they were not relevant to ENT.

### Article characteristics

Retracted studies were produced by research groups originating from 33 countries. The articles were originally published in 115 journals and were retracted between 1992 and 2023.

China produced the most retractions (29%), followed by the United States (20%), United Kingdom (11.1%) and South Korea (8%). Excluding South Korea, which appears to have a higher rate of retraction, this corresponds with the publication rates among these three countries [7]. The remaining countries produced less than 5% of all retractions. One author contributed to 7% of all retractions as either primary or secondary author, but this was an exception since retractions mostly derived from different authors.

Retracted literature was most often translational in design (*n* = 87, 38.7%). This was followed by prospective cohort studies (*n* = 22, 9.8%), clinical case reports (*n* = 16, 7.1%), systematic reviews with meta-analysis (*n* = 16, 7.1%), Randomised Controlled Trials (RCTs) (n = 16, 7.1%), narrative literature reviews (*n* = 16, 7.1%), retrospective cohort studies (*n* = 13, 5.8%) and systematic reviews without meta-analysis (*n* = 13, 5.8%). Case control studies (*n* = 6, 2.7%), cross-sectional studies (*n* = 4, 1.8%), quasi-randomised trials (*n* = 3, 0.9%), case series (*n* = 1, 0.4%) and letters to editor (*n* = 1, 0.4%) accounted for even fewer retractions. We could not discern the research design of 10 studies as the title and abstracts were too ambiguous.

Moreover, 80 (35.6%) studies pertained to head and neck, 66 (29.3%) to rhinology, 64 (28.4%) to otology and 13 (5.8%) to paediatric subspecialty. Two nursing studies did not meet the classification.

### Trends in retracted articles

**Fig. 2:**
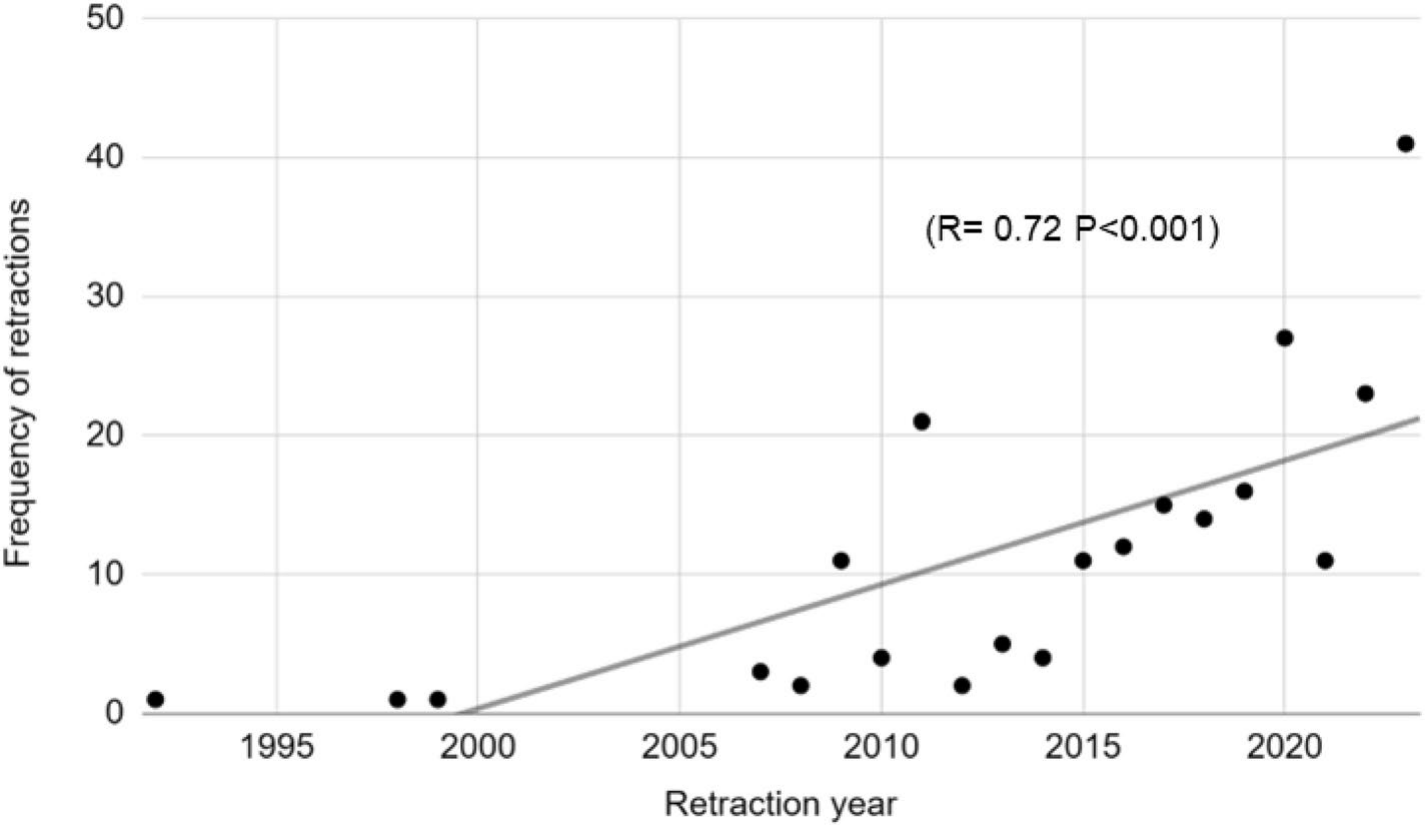
Scatter plot demonstrating the overall increase in frequency of retracted articles between 1992 and 2023

Overall, the frequency of retractions increased between 1992 and 2023 (Fig. 2). Time in years was a significant predictor of retraction frequency in regression analysis (*R*^2^ = 0.5, *F* = 18.1, *p* < 0.001). The median time from publication to retraction was 1 year, ranging from 0–29 years. The median impact factor of publishing journals was 3.2 (range: 0.02–168). The Lancet journal had the highest impact factor of 168.

### Method of signposting retraction

Overall, 191/225 retracted articles had a formal retraction notice. Among the publications signposted with retraction, 158 were PubMed-indexed and 4% of PubMed entries failed to reproduce the publisher’s retraction notice. We also explored whether retraction notices complied with COPE guidance, which states that retraction notices must be clearly identifiable and linked to the original publication. In our data, 44.4% of retraction notices were linked to a manuscript watermarked ‘retracted’ (Table 1). Fewer articles had a retraction notice linked to an article containing no watermark (22.2%). A minority of the manuscripts were removed but the retraction notices were present (10.2%).

**Table 1:** Method of signposting article retraction

	Frequency *N* (%)
Linked retraction notice and watermark label	100 (44.4)
Retraction notice linked to publication	50 (22.2)
Retraction notice unlinked to publication	18 (8)
Retraction notice and article removed	23 (10.2)
Link to updated review	23 (10.2)
No retraction notice	7 (3.1)
Abstract and paper unavailable	4 (1.8)

We classified systematic reviews with and without meta-analysis separately, as all were Cochrane reviews and did not have retraction notices but contained a link to the updated review (10.2%) (Table 1). Outdated reviews could be sourced in the updated review’s version history but were unavailable on PubMed. Four abstracts and manuscripts were unavailable to appraise. In our dataset, only seven publications had no retraction notice at all. Additionally, 15.1% of the retracted literature was behind a paywall, and although the manuscript required a subscription, the retraction notice was accessible.

### Causes of article retraction

The most common reason an article was retracted was due to duplication (26.7%), followed by multiple reasons (16.9%) and data error (12.9%) (see Table 2). A minority of research was retracted due to peer review manipulation (5.8%), falsified data (5.8%), and lack of Institutional Review Board (IRB) approval (4%). Seven retraction notices omitted the reason for retraction. Multiple reasons included one or more types of misconduct, including peer review manipulation, falsified data, duplication and lack of IRB. Moreover, dichotomising the cause for retraction into misconduct Vs other reasons revealed this misconduct accounted for most retractions (72%). Fewer articles were retracted for non-misconduct (23.1%) i.e. genuine errors, replacement with an updated review, or administrative causes such as copyright.

**Table 2:** Reason for retraction

	Frequency * N *(%)
Duplication	60 (26.7)
Multiple reasons	38 (16.9)
Data errors	29 (12.9)
Plagiarism	22 (9.8)
Replacement with updated review	20 (8.9)
Falsified data	13 (5.8)
Peer review manipulation	13 (5.8)
IRB	9 (4)
No reason	7 (3.1)
Author dispute	6 (2.7)
No information as no retraction notice	4 (1.8)
Copyright	3 (1.3)
Articles retracted due to poor reputation of author	1 (0.4)

*IRB* institutional review board

### Years-till-retraction

We also explored potential factors associated with years-till-retraction to explore the transparency of journals managing retractions. Years-till-retraction was shorter in more recent publications (RS = –0.57, *p* < 0.001), suggesting that journals are more expeditious in processing retractions. When stratified by cause of retraction, publications with misconduct had a longer time-to-retraction in years (*Z* = –2.6 *p* = 0.009). We also found impact factor of publishing journal positively correlated with retraction time (RS = 0.41, *p* = 0.001).

### Citation analysis

The number of citations following retraction was higher relative to the average citation counts prior to retraction (median 2, range 0–75 vs median 0, range 0–215), but this was not statistically significant (*Z* –1.78, *p* = 0.075). We sought to explore potential barriers to post-retraction citation, and found that paywall (*p* = 0.804), the presence of retraction notice (*p* = 0.221), and the cause of retraction made no difference (*p* = 0.087).

We also explored temporal trends in citation pattern. The number of citations received by articles was positively correlated with years-till-retraction, both before (RS 0.67, *p* < 0.001) and after retraction (RS 0.21, *p* = 0.002). Impact factor was positively associated with citation counts pre retraction (RS 0.4, *p* < 0.001) and after retraction (RS 0.36, *p* < 0.001). Additionally, the older the publication, the more citations it received before (RS –0.35, *p* < 0.001) and after retraction (RS –0.24, *p* < 0.001).

## Discussion

Retracted literature presents a challenge to the scientific community. In this systematic review of retracted ENT research, we have identified 225 retracted articles published between 1992 to 2023. We found the incidence of retraction was variable worldwide, arising from different authors, journals and countries, though the proportion of published papers retracted appeared similar between most countries.

Overall, retractions are rare and constitute only 0.02% of publications in PubMed; however, the issue is becoming more relevant as they have increased ten-fold in the last decade [3, 8, 9]. This review has substantiated that the number of retractions has risen over time for ENT. The literature also demonstrates the rise in retractions is more pronounced than the surge in publications, but there is still a lack of consensus on whether this represents an increase in incidence or detection of erroneous literature [3].

We investigated retraction time and impact factor of publishing journals to test the hypothesis that retractions have risen due to increased research visibility and scrutiny. In this instance, we could expect high-impact factor journals to retract articles faster than low-impact factor journals, as high-impact factor journals generate greater readership and citations. Interestingly, we observed the opposite: impact factor positively correlated with time-to-retraction. There are several explanations for this: perhaps high-impact journals are notified of retractable content later, or more thoroughly investigate such content, or proactively reach back to retract material. Steen et al., who studied 714 PubMed retractions concluded the trend in retractions represents a true increase in incidence of flawed research combined with an increase in detection [3]. It is possible the expanding reasons for retraction and the emergence of the internet have permitted scholars to recognise more fallacious data [3, 10]. Moreover, the internet has also enhanced plagiarism and image-duplication detection software available to publishers investigating retractions [10, 11].

The US Office of Research Integrity in the Department of Health and Human Services, COPE and the Retraction Watch, have all sought to raise awareness of retraction and encourage accountability and research integrity among journals and researchers alike [2, 10, 11]. A survey conducted by COPE in 2014 gave an insight into the early reluctance of publishers to retract articles; some believed authors were responsible for retraction; others were afraid of litigation [11, 12].The research paradigm has since started to change, with a growing attention to the veracity of medical research and the issue of retraction [1, 13–15]. Our data would support that journals are more vigilant to scientific misconduct and mistakes. First, ENT articles were retracted, on average, 1 year after publication. This is superior to the average three years-to-retraction of PubMed publications reported by Steen et al. [3]. Second, journals appeared to retract ENT literature faster in recent publications, as time-to-retraction negatively correlated with the publication year of retracted articles (–0.57, *p* < 0.001). This validates the trend of retracted PubMed literature: Steen et al. reported that the time-to-retraction was 49.8 months between 1973 and 2002, and only 23.8 months after 2002, and Furman et al*.* reported that the retraction time has decreased in the last three decades [3, 16].

Irrespective of whether erroneous articles are retracted efficiently, these articles have the potential to mislead academics and the general population from the moment of publication onwards, including after retraction. We evaluated whether journals met the COPE criteria that stipulate all retractions must be clearly signposted with a freely accessible retraction notice [4]. In the presence of a paywall, a subscription fee is required to access the manuscript, and readers may not be notified of retraction. In this review, there were 15.1% paywalled articles but the retraction notice was still freely accessible, although the manuscript was not. We encountered a variety in retraction notice practices, as previously described [1, 11, 17] ranging from retraction notices linked to a watermarked article, and retraction notices alone with the original article removed. Most retraction notices were linked to the publication and only 3.1% of manuscripts omitted a retraction notice entirely. This is superior to other studies of PubMed indexed retractions where retraction notices were absent in 22% of retractions [18].

We also evaluated whether retraction notices fulfilled additional COPE criteria by clearly specifying the reason for retraction. One publisher’s retraction statement included that retraction could be due to one or more of several listed reasons. We classified these six retractions as due to ‘multiple reasons,’ and acknowledge this may have overinflated that category. Previous studies found publisher notifications of misconduct were ambiguous compared to those for genuine errors [1, 19]. Reviews of retracted surgical literature and PubMed indexed retractions found 8.7% and 11% of notices respectively, did not disclose the cause of retraction [14, 20]. In contrast, this review found the vast majority (96.9%) of retraction notices were clear in specifying the reason for retraction. Our findings that most journals include a freely accessible retraction notice that declares a cause of retraction, supports the notion that there is greater transparency among journals.

Furthermore, this review revealed that 72% of ENT publications were retracted due to misconduct. This figure is comparable with the 82.7% of orthopaedic and 77.4% biomedical publications retracted due to misconduct [13, 21]. A review of retracted surgical literature grouped causes by administrative and content related instead, thus limiting comparison with our review. Evaluating the individual causes of retractions validated our findings that duplication was the most frequent cause (35.3%) [14]. In contrast, earlier studies advocate that honest error is more prevalent than misconduct [20, 22]. These studies may differ because they were conducted 1–2 decades ago and relied on PubMed and publisher retraction notices. We sourced most retracted articles (169/225) from the Retraction Watch—an arguably more comprehensive and objective source of retractions [11]. We also found seven retraction notices from the Retraction Watch that were not publicised on PubMed with the original manuscript. Another explanation for the difference in our results is the longer time-to-retraction we observed for misconduct compared to non-misconduct (Z = –2.6 p = 0.009). Steen RG et al*.* and Nath SB et al*.* corroborated that misconduct is associated with longer retraction time than honest errors [3, 20]. This trend likely reflects the significant time a publisher requires to review potential misconduct in order to be sure that a retraction is necessary. Therefore, it is possible that recent studies of retraction, including ours, are capturing the lag of retractions due to misconduct.

Retracted articles often continue to impact the literature via ongoing citation [5, 23]. Noorden et al*.* showed that 235 retractions between 1966–1996, were cited 2000 times after their withdrawal [10]. There are also estimates, based on a range of studies spanning the last 20 years, that over 50% of literature citing retracted articles accept the original evidence [15, 16, 24–26]. We thought it was imperative to explore this, given the potential impact on medical research, particularly with inclusion in systematic reviews. We found that articles continued to be cited after retraction, indeed even more than prior to retraction (median 2 vs 0, *Z* –1.78, *p* = 0.075). Citation counts positively correlated with time-to-retraction and journal impact factor; this was expected since impact factor is determined by the journal’s citation index.

Interestingly, the presence of retraction notices did not impact citation rate, but a shorter time from publication to retraction did reduce it, highlighting the need for journal vigilance and action. Overall, reassuringly, the median citation count was low. Whereas our study limits its citation analysis to correlation, Furman et al*.*, used case-matched controls to investigate citations of 677 biomedical retractions. The authors found a 50% decrease in citations two years after retraction, and a sustained decrease to 72% compared to non-retracted work by the tenth year [16].

Interestingly, there is evidence that there is a citation penalty for authors of work retracted due to misconduct, causing a decline in citations of subsequent articles produced by the author [27]. In our review, one study was retracted, not based on its own credibility, but as part of a larger group of publications by the same author. This supports the hypothesis that journals may appraise other articles by the author previously penalised by retraction. However, the research also concluded that there was no citation penalty for authors that self-report errors or misconduct [27]. The Retraction Watch editors encourage authors to be transparent and concur that honest errors will not be detrimental to future research careers [11, 20]. The consequences appear more deleterious to the public, as Steen et al*.* found that 9,189 patients were enrolled into 180 RCTs that were later retracted [5]. Taken together, the balance of evidence would favour being open and honest in disclosing any mistakes or fraud to publishing journals. Having a forthcoming approach lies in the public’s best interest, and the impact on researchers is minimal provided they demonstrate integrity.

## Limitations

We cannot capture the studies that will be subject to future investigation and later retraction, and there could be a 1–3-year lag in retractions based on our data and previous scholarship [3]. Moreover, this was a limitation of calculating years-till-retraction as a measure of retraction efficiency. There is also evidence that it may take a further 3 years for the retraction notice to appear on PubMed [28]. When evaluating the trends of retraction, our conclusions were limited because we were unable to obtain the journal statistics needed to calculate the rate of retraction. Although we could have conducted two separate analyses for EMBASE and MEDLINE retractions, most retractions were from the Retraction Watch. Furthermore, our citation analysis could be improved by qualitative analysis of citing articles to explore if the citing authors accept original conclusions or acknowledge the retraction. This has not been performed in clinical or surgical literature to the best of our knowledge, and future studies could benefit from this.

## Conclusion

Overall, retraction is a rare event in the medical community, but our findings suggest that journals are transparent in identifying and publishing these promptly in the ENT literature when compared to other areas of medicine. Clinical researchers should be cautious when conducting their own research and critically appraise manuscripts before citing literature to avoid the ongoing citation of retracted and possibly fraudulent work. We share our findings to draw attention to retraction in ENT literature and encourage vigilance towards potential fraudulent and erroneous research that could jeopardise medical advances and patient care.

## Supplementary Information

Below is the link to the electronic supplementary material.

Supplementary file1 (DOCX 7 kb)

## Data Availability

The data that support the findings of this study are available from the corresponding author upon reasonable request. Additional data that supports the findings of this study are available in the supplementary material of this article.
