# Correction: A systematic review of ENT retractions

**DOI:** 10.1007/s00405-024-09119-5

**Published:** 2024-12-30

**Authors:** Rosalind Di Traglia, Henry Dunne, James Tysome, Matthew E. Smith

**Affiliations:** 1 School of Clinical Medicine, University of Cambridge, Cambridge, UK; 2 Cambridge University Hospitals NHS Foundation Trust, Cambridge, UK; 3 Department of ENT, Addenbrookes Hospital, Cambridge University Hospitals NHS Foundation Trust, Cambridge, CB2 0SP UK

**European Archives of Oto-Rhino-Laryngology**

10.1007/s00405-024-08980-8

In this article ref. ‘Marcus A, Oranksky I (2014) What studies of retractions tell us. J Microbiol Biol Educ 15:151–154. 10.1128/jmbe. v15i2.855’ was incorrect and should have been' Marcus A, Oransky I (2014) What studies of retractions tell us. J Microbiol Biol Educ 15:151–154. 10.1128/jmbe. v15i2.855’.

The original article has been corrected.

