# Supplementary material for: A systematic review of ENT retractions
Source: Eur Arch Otorhinolaryngol. 2024 Oct 14;282(2):1041–8. doi: 10.1007/s00405-024-08980-8 (PMC11805887; doi:10.1007/s00405-024-08980-8)
Supplement: Supplementary file 1 — Supplementary file1 (DOCX 7 kb) [file 405_2024_8980_MOESM1_ESM.docx]

**Systematic Searches**

|  | MEDLINE |
| --- | --- |
| #1 | (ear or nose or throat).ti,ab,kw,kf. |
| #2 | otolaryngology.ti,ab,kw,kf. |
| #3 | exp Otolaryngology/ |
| #4 | Otorhinolaryngology.ti,ab,kw,kf. |
| #5 | Ear Diseases/ |
| #6 | Pharynx/ |
| #7 | Nose Diseases/ |
| #8 | ENT.ti,ab,kw,kf. |
| #9 | (head or neck) .ti,ab,kw,kf. |
| #10 | 1 OR 2 OR 3 OR 4 OR 5 OR 6 OR 7 OR 8 OR 9 |
| #11 | limit 10 to (retracted publication or “retraction of publication” |

|  | EMBASE |
| --- | --- |
| #1 | *ear/ or ear.ti,ab. |
| #2 | *nose/ or nose.ti,ab. |
| #3 | *throat/ or throat.ti,ab. |
| #4 | *otorhinolaryngology/ or ortorhinolaryngology.ti,ab. |
| #5 | *ear nose throat disease/ or *throat disease/ |
| #6 | *ear disease/ or *inner ear disease/ or middle ear disease/ |
| #7 | ear disease.ti,ab. |
| #8 | "head and neck disease"/ or "head and neck infection"/ |
| #9 | *"head and neck carcinoma"/ or *"head and neck metastasis"/ or *"head and neck tumor"/ |
| #10 | *"head and neck surgery"/ |
| #11 | (head or neck).ti,ab. |
| #12 | 1 or 2 or 3 or 4 or 5 or 6 or 7 or 8 or 9 or 10 or 11 |
| #13 | limit 12 to retraction notice |
